# Pharmaceutical Strategic Purchasing: a Key to Improve Access to Medicines 

**Published:** 2015

**Authors:** Gholamhossein Mehralian, Peivand Bastani


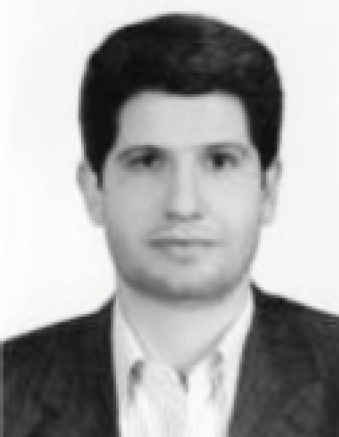


Pharmaceuticals are often considered as the most significant cost driver of health care expenditures and at the same time have a major impact on patients’ curative processes and outcomes. Other pieces of evidence emphasize that drugs are the input for the production of good health. They are considered as the most important and expensive element in the healthy supply chain and are more associated with the management and economics rather than other factors. So, the availability of the relevant, effective, and payable drugs at the time of need and their rapid diffusion at affordable prices are important indicators of the utilization of health services. They can be considered as the most visible determinant of quality and the major driver source of the phenomenal increase in longevity of the human race over the past decades.

 World Health Organization data implies that most of the spending on pharmaceuticals in developing countries is privately financed (between 45 and 90 percent of all drug expenditures) and a large part of household health care expenditures is spent on pharmaceuticals. As some evidences indicate, despite the growth of the pharmaceutical production and distribution industry, and achieving the self-sufficiency boundaries in the country, patients’ out of pocket for drugs are around 65% which can seriously restrict their ability to pay for useful drugs. Literature shows that increased access to drugs depends on effective and collective resource allocation, an efficient purchasing system, rational selection and use of medicines, adequate and sustainable financing, affordable prices, and reliable health and supply systems. Among these factors, employing an efficient purchasing system is known as a strategic purchasing, opposed to a passive purchasing, with the allocation of an obvious budget or payment of a predetermined bill; such a system is applied in many developing countries right now.

In recent years, demand for strategic purchasing has increased which has resulted in a shift towards centralized purchasing organizations and an increase in long term contracts over spot buying. The strategic options to improve drug access entail measures stimulating optimal functioning of the market through incentives, regulations, and information in a context of strategic purchasing. These strategies may largely be classified into demand side interventions, supply side interventions, pricing, and incentives. 

In other words, there are five questions which should be answered before any strategic purchasing: 

1- For whom should it be bought? Specifying the groups that have a greater need for medicines and allocating available subsidies to increase their access to essential drugs considering the rational use, applying pharmaceuticals for high-priority services (*e.g*., maternal and child health, family planning, elderly, *etc*.), and for diseases of public health importance (*e.g*., tuberculosis, HIV, and so on). 

2- Regarding cost effectiveness, safety, quality, and price, which medicine should be bought?(Emphasizing on purchasing essential drugs and generic drugs rather than brands). 

3- From whom should the drug be bought? Determining the best and more qualified pharmaceutical suppliers, given that the selection of drugs and providers is a key issue for resource allocation and strategic purchasing. 

4- At what price should it be bought? Increasing the affordability of drugs requires that purchases be made at the lowest prices for the same standard quality. Such a situation can be reached only in a fair competition context. In other words, prices may be decreased through more competitive procurement by centralized procurement agencies (international tendering) or even by own procurement (local tendering). 

5- How should it be paid? Paying attention to the nature of pharmaceutical market that has congenital information asymmetries, a combination of a relatively competitive retail market, irrational prescription, overpricing, and unequal distribution is required to apply appropriate incentives especially in a payment system, regulatory mechanisms, and penalties.

Pieces of evidence show that, to put these theories into practice, larger resource allocation and purchasing agents should be able to exert collective consumer influence over large providers thereby make more effective purchases ensuring high-quality services and drugs at affordable prices. Moreover, there are some successful examples of developing countries in the world which has implemented pharmaceutical strategic purchasing with the integrated health care. 

*Gholamhossein Mehralian is currently working as assistance at the Department of Pharmacoeconomic and Pharma management, School of Pharmacy, Shahid Beheshti University of Medical Sciences, Tehran, Iran. He could be reached at the following e-mail address:gmehralian@gmail.com*

